# Supplementary material for: Targeted seed EMS mutagenesis reveals a basic helix–loop–helix transcription factor underlying male sterility in sorghum
Source: Genetics. 2025 Jan 30;230(3):iyaf017. doi: 10.1093/genetics/iyaf017 (PMC12239208; doi:10.1093/genetics/iyaf017)
Supplement: iyaf017_Supplementary_Data [file iyaf017_supplementary_data.pdf]

Table S1. DNA sequences used in generating and genotyping *ms8* CRISPR knockout lines.

Name	Sequence (5'-3')	Purpose
gRNA1	CCTTACCTCTGAGCCTGCAA	gRNA sequence 1
gRNA2	AGGTCCACATAACATAACTG	gRNA sequence 2
gRNA3	GGAGTAGCAACTCATGAAAG	gRNA sequence 3
gRNA4	AGTTCGACAGCCCAGTACTG	gRNA sequence 4
ms8_CRS_F1	TATCTTGAGTGAAGGTAACCAGG	primer to genotype the CRISPR lines
ms8_CRS_R2	TTAGTAGGGTTTGGGAACAGTG	primer to genotype the CRISPR lines

Table S2. Basic statistics of *ms8-1* BSA-seq libraries.

DNA pool	Panicle phenotype	No. of samples	Reads	Mapped reads	% Mapped reads	Mapped reads after filtering	Coverage
ms8_pool1	male sterile	57	91,838,342	88,430,940	96.29%	55,299,475.00	13.03
wtsib_pool1	wildtype-like	40	101,935,608	98,026,304	96.16%	60,940,906.00	14.32
ms8_pool2	male sterile	37	62,420,472	60,149,061	96.36%	37,215,222.00	8.88
wtsib_pool2	wildtype-like	42	62,923,504	60,336,095	95.89%	37,283,383.00	8.91

Table S3. Summary of homozygous variants detected in *ms8-1* mutant pools.

Region	Variants	ms8_pool1	ms8_pool2
Genome-wide	Total number of homozygous variants	5711	4400
	Number of homozygous variants after filtering	48	101
Region of Interest (56-64Mbp on Chr4)	Total number of homozygous variants	52	59
	Number of homozygous variants after filtering	44	48
	Number of shared homozygous variants	28	

Table S4: Shared homozygous variants within the 56-64 Mbp region on chromosome 4 in the two *ms8-1* mutant pools.

CHROM	POS	REF	ALT	QUAL	DP	GT
Chr4	58313093	C	T	108	5	1/1
Chr4	58405041	C	T	205	11	1/1
Chr4	58776014	C	T	225	21	1/1
Chr4	58903008	C	T	135	7	1/1
Chr4	58963190	C	T	225	20	1/1
Chr4	59245995	C	T	208	11	1/1
Chr4	59331944	C	T	225	20	1/1
Chr4	59750334	C	T	225	23	1/1
Chr4	59857364	C	T	225	16	1/1
Chr4	59887012	C	T	212	11	1/1
Chr4	59914160	C	T	150	7	1/1
Chr4	59937306	C	T	87	7	1/1
Chr4	60000967	C	T	225	11	1/1
Chr4	60004891	C	T	69	4	1/1
Chr4	60155882	C	T	225	24	1/1
Chr4	60299380	C	T	205	13	1/1
Chr4	60333759	C	T	168	13	1/1
Chr4	60487987	C	T	218	13	1/1
Chr4	60661154	C	T	225	13	1/1
Chr4	61051131	C	T	225	22	1/1
Chr4	61193951	C	T	225	19	1/1
Chr4	61212202	C	T	201	16	1/1
Chr4	61220618	C	T	225	21	1/1
Chr4	61274109	C	T	225	21	1/1
Chr4	61465066	C	T	225	20	1/1
Chr4	61494009	C	T	225	20	1/1
Chr4	61731674	C	T	225	20	1/1
Chr4	61914899	C	T	216	10	1/1

Note: CHROM: Chromosome; POS: Position of the variant on the chromosome; REF: Reference base at this position; ALT: Alternate (mutant) base; QUAL: Confidence score for the variant call; DP: Read depth at this position; GT: Genotype.

Table S5. Sequencing summary statistics from the whole genome DNA sequencing of the male fertile and male sterile sectors.

Sample Name	Total QC passed reads	Reads mapped properly paired	Depth of Sequencing
MS8/ms8-1 male fertile sector	273684893	247614956	60
ms8-1/ms8-2 male sterile sector	280119689	259547270	61

Table S6. Summary of all candidate genes along with polymorphisms causing moderate to high impact protein functional change identified in whole genome sequencing of ms8-1 homozygous samples.

Gene Name	Position	Variant Type	SNP	***MS8*/*ms8*-1**<br>**(fertile sector)**	***ms8*-1/*ms8*-2**<br>**(sterile sector)**	
Sobic.004G253400	59,914,160	Missense variant	G>A	G: 15/32 (47%)<br>A: 17/32 (53%)	G: 24/37 (65%)<br>A: 13/37 (35%)	
Sobic.004G255500	60,155,882	Missense variant	G>A	G: 24/45 (53%)<br>A: 22/35 (63%)	G: 19/36 (53%)<br>A: 17/36 (47%)	
Sobic.004G270900	61,494,009	Nonsense variant	C>T	C: 13/35 (37%)<br>T: 22/35 (63%)	C: 29/62 (47%)<br>T: 33/62 (53%)	P98S and Q111*
Sobic.004G273500	61,731,674	Missense variant	C>T	C: 24/42 (57%)<br>T: 18/42 (43%)	C: 27/54 (50%)<br>T: 27/54 (50%)	
Sobic.004G275800	61,914,899	Missense variant	G>A	G: 13/20 (65%)<br>A: 7/20 (35%)	G: 11/24 (46%)<br>A: 13/24 (54%)	

Note: The heterozygous genotype at each SNP, along with read counts and allele frequency, in pollen-fertile and pollen-sterile sector is also shown.

Figure S1. Summary of the reads overlapping the *ms8-1* and *ms8-2* positions.

	ms8-2			***ms8-1***	***ms8-1/ ms8-2***
**All reads at position**	**C>T<br>P98S**	**C>T<br>Q111***	**P98S<br>and<br>Q111***	**C>T<br>Q150***	**Q111*<br>and<br>Q150***
MS8/ms8-1<br>(male fertile)	50 reads total<br>All reads Wt C	46 reads total<br>All reads Wt C	32 reads total<br>All reads Wt CC	35 reads total<br>13 reads Wt C<br>22 reads Mut T	11 reads total<br>5 reads *ms8-1* CT<br>6 reads Wt CC
ms8-1/ms8-2<br>(male sterile)	48 reads total<br>17 reads Wt C<br>31 reads Mut T	52 reads total<br>21 reads Wt C<br>31 reads Mut<br>T	36 reads total<br>13 reads Wt CC<br>23 reads Mut TT<br>No mixed reads	62 reads total<br>29 reads Wt C<br>33 reads Mut T	14 reads total<br>7 reads *ms8-1* CT<br>7 reads *ms8-2* TC<br>No Wt reads<br>No mixed reads

Note: The novel *ms8-2* allele consists of two C>T transitions linked in cis resulting in P98S and Q111* changes that are exclusive to the male-sterile sector and fail to complement *ms8-1* allele that carries a C>T transition resulting in a Q150* nonsense variant.
